# Evolution and contemporary landscape of neonatal hyperbilirubinemia management guidelines: a narrative review

**DOI:** 10.3389/fped.2026.1745769

**Published:** 2026-02-06

**Authors:** Yaxin Zhang, Yaowen Zhang, Qin Huang, Shuhua Yi, Xiaoqing Guan, Hongyu Li, Jun He

**Affiliations:** 1 Department of Obstetrics and Gynecology, The First Affiliated Hospital of the Army Medical University, Chongqing, China; 2 Department of Pediatrics, The First Affiliated Hospital of the Army Medical University, Chongqing, China

**Keywords:** clinical practice guidelines, contextual adaptation, global harmonization, global health disparities, neonatal jaundice, standardized management

## Abstract

Neonatal hyperbilirubinemia, affecting over 60% of term and 80% of preterm infants globally, remains a significant public health challenge due to the persistent risk of kernicterus despite effective management strategies. This narrative review summarizes global clinical practice guidelines to describe the evolution and current landscape of management, highlighting the disparity between evidence-based consensus and local adaptation. While universal principles such as early hyperbilirubinemia screening, phototherapy as the first-line treatment, and post-discharge follow-up are widely endorsed, substantial disparities exist in implementation—particularly between high-income countries and low- and middle-income countries (LMICs)—driven by differences in healthcare resources, sociocultural perceptions, and technological access. The 2025 Chinese guideline is an integrated model combining risk-stratified screening, locally derived hour-specific nomograms, online follow-up, and family engagement, offering a scalable framework for LMICs. Looking ahead, global harmonization may require a dual-layered guideline structure, affordable technology dissemination, culturally adapted family support systems, and inclusion of hyperbilirubinemia metrics in national health performance evaluations to achieve equitable and effective care worldwide.

## 1 Introduction

Neonatal hyperbilirubinemia remains one of the most prevalent clinical conditions worldwide, affecting approximately 60% of term infants and up to 80% of preterm infants within the first week of life (1). Over the past three decades, the global burden of neonatal hyperbilirubinemia has shifted substantially. According to the Global Burden of Disease Study, between 1990 and 2019, advances in screening and management were associated with a nearly 60% reduction in mortality attributable to hyperbilirubinemia (2). Nonetheless, pronounced inequities persist between high-income countries and low- and middle-income countries (LMICs). In resource-limited settings, neonatal hyperbilirubinemia remains a leading cause of preventable neonatal mortality and disability, which may stem from systemic inequities in bilirubin measurement technologies, accessibility to phototherapy equipment, and caregiver health education (3).

In response, countries worldwide, along with the World Health Organization (WHO), have developed and periodically updated evidence-based neonatal hyperbilirubinemia management guidelines (3–8), emphasizing early recognition, risk stratification, standardized treatment thresholds, and parental engagement. However, due to variations in healthcare system structures, resource allocation, and cultural perceptions, significant differences remain among countries regarding these issues (8–10). Despite the global convergence of principles in neonatal hyperbilirubinemia management, international disparities in guideline implementation highlight the importance of standardized collaboration and knowledge sharing (11, 12).

We searched major bibliographic databases—PubMed/MEDLINE, Embase, and Web of Science—from 1992 to 2025. The search strategy employed combinations of keywords such as “neonatal hyperbilirubinemia”, “neonatal jaundice” and “clinical practice guideline.” To ensure comprehensive coverage, we also performed manual searches of the official websites of the World Health Organization, national pediatric societies, and relevant health authorities. Inclusion criteria comprised authoritative clinical practice guidelines and consensus statements addressing the diagnosis or management of neonatal hyperbilirubinemia, available in English or Chinese. Exclusion criteria included local institutional protocols, outdated guideline versions, and non-official publications. By systematically comparing the evolution and core content of national guidelines, we analyzed the underlying policy, resource, and cultural drivers. We identified best practices and implementation gaps, with particular emphasis on the innovations and international relevance of China's 2025 guideline. We further discuss opportunities and challenges for global harmonization and propose evidence-based recommendations and implementation strategies to strengthen standardization of neonatal hyperbilirubinemia diagnosis and treatment and to advance global health equity (8).

## 2 Evolution and core consensus in hyperbilirubinemia management

The management of neonatal hyperbilirubinemia has evolved from largely empirical, experience-based practice to the adoption of evidence-based and standardized protocols. Initially, clinical assessment relied largely on healthcare providers' subjective evaluation of skin discoloration, a method prone to error because of variations in lighting, skin pigmentation, and individual clinical experience, thereby increasing the risk of both misdiagnosis and underdiagnosis (13). Advancements in bilirubin measurement technologies, notably the widespread implementation of total serum bilirubin (TSB) and transcutaneous bilirubin (TcB) assessments, have markedly enhanced early identification and risk-stratified management of hyperbilirubinemia. These developments have laid a robust foundation for determining timely intervention thresholds and therapeutic pathways (14).

**Figure 1:**
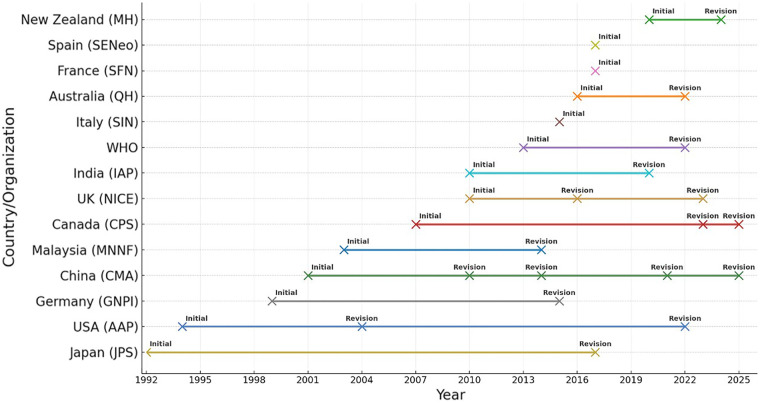
Global neonatal hyperbilirubinemia guideline publication and revision timeline (1992–2025).

Since the 1990s, numerous countries and authoritative international organizations have established and refined neonatal hyperbilirubinemia management systems, steering global practices toward a paradigm characterized by evidence-based medicine, risk stratification, and standardized procedures (3–8), as shown in Figure 1.

The American Academy of Pediatrics (AAP) introduced its inaugural guidelines for managing neonatal hyperbilirubinemia in 1994 (15). In 2004, the AAP incorporated the Bhutani nomogram to facilitate dynamic bilirubin assessment based on postnatal age in hours (16). The most recent revision in 2022 emphasizes an integrated approach combining gestational age–specific thresholds, tiered interventions, and parental education (4). The United Kingdom's National Institute for Health and Care Excellence (NICE) released its neonatal hyperbilirubinemia management guideline in 2010, with updates in 2016 and 2023 that emphasize universal screening protocols, standardized phototherapy criteria, and detailed post-discharge follow-up (5). The WHO issued recommendations in 2013 and revised them in 2022 (6), aiming to balance feasibility and safety across diverse resource settings and promote structural consistency in global guideline. China's neonatal hyperbilirubinemia guidance has evolved from expert recommendations in 2001 and consensus statements in 2010, 2014, and 2021 to an evidence-based, standardized 2025 guideline (7). This progression reflects gradual alignment of China's domestic management approach with international standards, while incorporating localized innovations tailored to its multi-tiered health system and high-risk population profiles.

Despite variations in implementation timing, guideline scope, and healthcare system contexts, the overall trajectory of guideline evolution has been strikingly consistent (3–8). Across regions, successive updates reveal a clear convergence toward objective bilirubin measurement, structured risk stratification, and proactive prevention of bilirubin-induced neurologic injury. Grounded in these shared principles, countries tailor screening strategies, intervention thresholds, therapeutic pathways, and follow-up frameworks to their health-system infrastructure, sociocultural context, and resource capacity. This pattern highlights a defining feature of contemporary guideline development: the pursuit of standardization while maintaining contextual flexibility.

## 3 Narrative synthesis of major guidelines

As the burden and management needs of neonatal hyperbilirubinemia have evolved (17), countries and international organizations have iteratively updated their guidelines in response to local healthcare resources, demographic profiles, and epidemiologic patterns. Major global guidelines typically comprise five domains: screening and diagnosis; risk assessment and intervention thresholds; treatment pathways and monitoring; post-discharge follow-up and family engagement; and management of high-risk populations. However, substantive heterogeneity persists: specific design choices and implementation details reflect the diversity and adaptability of healthcare systems, policy priorities, and resource availability, as detailed in Table 1.

**Table 1:** Comparative analysis of guideline structures and core recommendations.

Country/Organization	Bilirubin Assessment Tools	Phototherapy Initiation Criteria	Management of High-Risk Infants	Discharge and Follow-Up Arrangements
China (7)	TcB + TSB; hour-specific nomogram	Stratified by gestational age, postnatal age, and risk factors	Enhanced screening for glucose-6-phosphate dehydrogenase (G6PD) deficiency and infections	Follow-up within 48–72 h post-discharge; promotion of app-based follow-up
United States (4)	TcB + TSB; AAP hour-specific nomogram	Stratified by hour-specific nomogram; consider neurotoxicity risk factors	Focus on hemolysis, G6PD deficiency, prematurity	Re-evaluation within 48–72 h post-discharge; provide parental education materials
United Kingdom (5)	TcB (≥35 weeks gestation) + TSB	Hour-specific and concentration-based thresholds	Emphasis on prematurity, low birth weight, breastfeeding	Community nurse visits; family education guidance
WHO (6)	Primarily TSB	Simplified threshold charts suitable for resource-limited settings	Provision of rapid assessment checklist for high-risk infants	Follow-up recommended within 72 h post-discharge
Japan (18)	TcB + TSB	Lower thresholds for preterm infants; hour-specific nomogram	Dedicated management chapter for infants <35 weeks gestation	Comprehensive follow-up system; emphasis on family involvement
Canada (19)	TcB + TSB	Similar to AAP; hour-specific nomogram	Individualized assessment model incorporating multiple risk factors	Unified tracking system; emphasis on parental education
Germany (20)	TSB	Traditional reference values; hour-specific nomogram not utilized	Partial mention of high-risk populations	Follow-up recommended; specific timing not detailed
Italy (21)	TSB	Traditional reference values; hour-specific nomogram not utilized	Partial mention of high-risk populations	Post-discharge follow-up arrangements not explicitly stated
Spain (22)	TcB + TSB	Hour-specific and concentration-based reference values	Partial mention of high-risk populations	Emphasis on family recognition and follow-up
India (23)	TSB	Relatively broad thresholds adapted to resource availability	Emphasis on G6PD screening and management	Follow-up recommended; implementation varies due to resource constraints
Australia (24)	TcB + TSB	Hour-specific thresholds; specific criteria provided	Enhanced pre-discharge assessment; focus on high-risk factors	Availability of home phototherapy; emphasis on mother-infant interaction
Malaysia (25)	Primarily TSB	Resource-adapted thresholds considering equipment accessibility	Partial high-risk management; emphasis on mother-infant interaction	Emphasis on mother-infant interaction; follow-up recommended
New Zealand (26)	TcB + TSB	Reference charts; hour-specific nomogram	Detailed management protocols for preterm infants	Home-based follow-up mechanisms; emphasis on community support

### 3.1 Screening and diagnostic strategies

Virtually all guidelines recommend measuring total bilirubin (TB) levels within the first 24 h after birth. Countries exhibit significant differences in the scope, timing, and methods of neonatal hyperbilirubinemia screening, which can be broadly categorized into two approaches:

#### 3.1.1 Universal screening

Countries such as the United States, Canada, China, the United Kingdom, Japan, New Zealand, and Australia recommend bilirubin screening for all newborns within 24 h after birth or before hospital discharge. TcB measurement is preferred as the initial noninvasive screening tool, with TSB measured when TcB values approach treatment thresholds or in high-risk situations. This strategy aims to reduce missed cases by ensuring early detection across the entire newborn population.

#### 3.1.2 Risk-based screening

In countries such as India, Malaysia, and Italy, guidelines advocate targeted screening for newborns with clinical jaundice, prematurity, G6PD deficiency, or maternal–infant blood group incompatibility. These approaches reflect pragmatic adaptation to constrained resources, early discharge practices, and uneven access to laboratory testing, rather than disagreement with the principle of early detection.

Regarding screening tools, TcB is widely accepted as a first-line, noninvasive method, whereas TSB is used to confirm elevated levels and guide treatment decisions (27). China's 2025 guideline recommends initial screening 12–24 h after birth and emphasizes monitoring bilirubin trends. The WHO underscores the necessity of at least one bilirubin assessment (using either TcB or TSB) before discharge and within the first 72 h of life.

### 3.2 Treatment initiation criteria and methods

Phototherapy is universally recognized as the first-line treatment for neonatal hyperbilirubinemia. High-intensity blue-light phototherapy—most commonly delivered using light-emitting diode (LED) devices—is recommended for most cases of moderate [typically TSB 15–20 mg/dL (257–342 µmol/L)] to severe hyperbilirubinemia [TSB >20 mg/dL (>342 µmol/L)], whereas exchange transfusion is generally reserved for extreme, rapidly progressive, or phototherapy-refractory cases (4, 7, 28–30). However, initiation thresholds, phototherapy intensity, and monitoring frequency vary across regions, reflecting differences in evidence interpretation, resource availability, and clinical care pathways.

#### 3.2.1 Initiation thresholds

Countries such as the United States, Canada, the United Kingdom, China, and Japan use hour-specific TSB nomograms to determine when to initiate phototherapy. For instance, the 2022 AAP guideline provides hour-specific TSB thresholds stratified by gestational age and neurotoxicity risk factors, reflecting updated understanding of bilirubin-induced neurotoxicity. In resource-limited settings, the WHO and some national guidelines (e.g., India) adopt fixed TSB thresholds or broader reference tables to accommodate practical constraints in primary-care facilities.

#### 3.2.2 Phototherapy equipment

High-intensity LED blue-light devices are widely used in high-income settings because of their stable irradiance, minimal heat generation, and long service life, making them the standard in many facilities. China's 2025 guideline also recommends LED phototherapy as the standard modality. However, in resource-constrained settings such as India and Malaysia, some facilities still rely on conventional fluorescent lamps. New Zealand and the United Kingdom recommend double-sided phototherapy when feasible to improve treatment efficiency.

#### 3.2.3 Monitoring and evaluation

Most guidelines recommend measuring TSB every 4–12 h after phototherapy initiation to assess treatment response and enable timely adjustments, because TcB measurements are unreliable during phototherapy. For infants with rapidly progressing conditions or ineffective phototherapy, prompt escalation of treatment or consideration of exchange transfusion is advised (4–7). For example, guidelines suggest monitoring TSB levels every 4–6 h in infants undergoing phototherapy until hyperbilirubinemia is controlled, followed by re-evaluation every 12–24 h (5).

Pharmacologic adjuncts and exchange transfusion. When phototherapy is insufficient or severe hyperbilirubinemia is confirmed by TSB, some national guidelines recommend pharmacologic adjuncts and/or exchange transfusion (31, 32). Phenobarbital is used in some settings, including China and the United States, as an alternative or adjunctive therapy when phototherapy alone is insufficient. Indications for exchange transfusion vary substantially across national guidelines. The United States and China specify criteria based on severe hemolysis, rapidly rising TSB levels, and neurologic signs. WHO and Indian guidelines emphasize considering exchange transfusion when signs such as altered consciousness or diminished reflexes are present.

**Figure 2:**
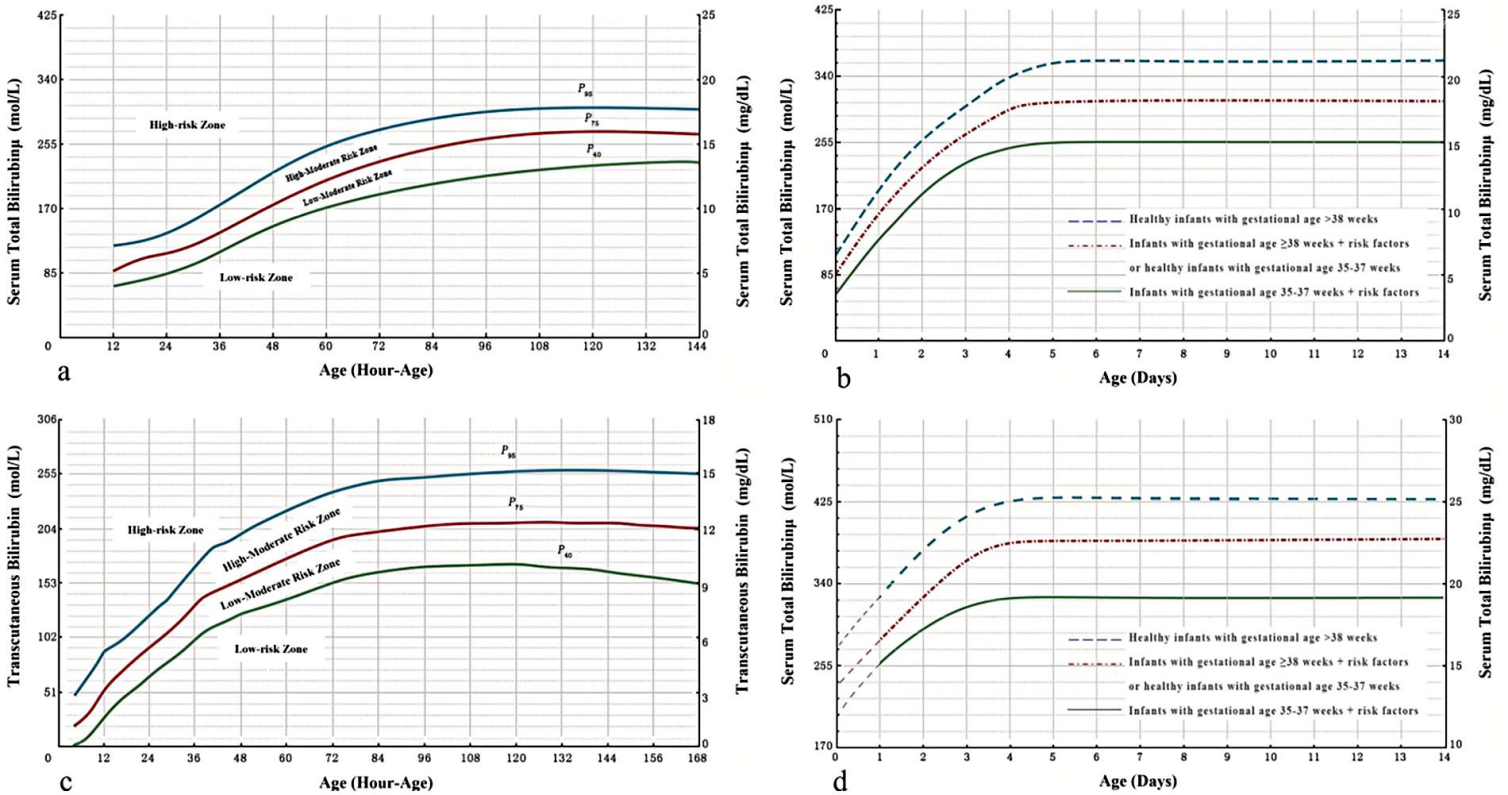
**(a)** Hour-specific nomogram of TSB levels for hyperbilirubinemia risk assessment in Chinese neonates; **(b)** hour-specific TSB–based phototherapy initiation thresholds for Chinese neonates with gestational age ≥35 weeks; **(c)** hour-specific nomogram of TcB levels for hyperbilirubinemia risk assessment in Chinese neonates; **(d)** hour-specific TSB–based exchange transfusion thresholds for Chinese neonates with gestational age ≥35 weeks.

Drawing on extensive national datasets, the 2025 Chinese guideline introduces hour-specific nomograms for both risk assessment and intervention thresholds (Figure 2; adapted from the 2025 Chinese guideline), reflecting a data-driven, localized approach to personalized management.

### 3.3 Management of high-risk populations

Most clinical guidelines advocate more intensive monitoring and treatment for specific high-risk neonates. Common risk factors include prematurity (gestational age <35 weeks), G6PD deficiency, maternal–infant ABO or Rh incompatibility, prolonged breast milk jaundice, and perinatal asphyxia, infections, and metabolic disorders.

China's 2025 guideline emphasizes routine G6PD screening and recommends assessing bilirubin trends (TSB or TcB, with TSB preferred to accurately quantify the rate of rise and to help exclude hemolysis) in high-risk infants within the first 12 h after birth. Japan has established dedicated management pathways for preterm infants (<35 weeks gestation), adopting lower TSB treatment thresholds. Canada employs a multilayered risk-stratification model that integrates TSB levels, gestational age, postnatal age (in hours), and additional risk factors. In regions with high G6PD prevalence (e.g., India), guidelines underscore the importance of screening and caution against trigger medications. The WHO provides a “rapid assessment checklist” tailored for resource-limited settings to minimize reliance on complex procedures.

### 3.4 Discharge criteria and follow-up arrangements

With the trend toward early discharge (most infants are discharged within 48 h after birth), post-discharge bilirubin reassessment has become a focal point in many national guidelines. The AAP, China, the United Kingdom, and Canada recommend bilirubin re-evaluation (TcB or TSB) within 48–72 h after discharge. Japan, New Zealand, and Germany advocate establishing community- or primary care–based follow-up systems. The WHO emphasizes that all infants discharged before 48 h should receive follow-up within three days.

Most guidelines advise providing caregivers with written, comprehensible materials on jaundice recognition, emergency contact information, and care instructions. China's 2025 guideline promotes home-based bilirubin monitoring devices and telemedicine platforms (e.g., mobile applications). Canada and New Zealand have developed integrated mother–infant follow-up systems to reduce missed cases. In Australia, New Zealand, and the United Kingdom, home phototherapy is increasingly used. China's 2025 guideline provides specific recommendations for primary care referral, home-based care, and follow-up assessment.

## 4 Drivers of variation in national practices

While international guidelines for neonatal hyperbilirubinemia management have converged on key principles—such as universal screening, phototherapy as first-line therapy, and post-discharge follow-up—substantial practice variation persists in initiation timing, treatment thresholds, management of high-risk populations, discharge protocols, and family-centered care. These differences reflect multiple determinants, including health-system structure, economic resource allocation, sociocultural contexts, and the methodological approaches used in guideline development (8).

### 4.1 Heterogeneity in healthcare system structures and resource allocation

Health-system organization and resource distribution directly influence the feasibility and implementation fidelity of guideline recommendations (33–35). High-income countries—such as the United States, Canada, the United Kingdom, China, and Japan—generally adopt universal screening strategies, supported by comprehensive maternal and child health networks, ample access to TcB and TSB testing resources, and standardized discharge assessment systems. By contrast, LMICs (e.g., India, Malaysia, Italy) often face challenges such as uneven healthcare resource coverage and a lack of phototherapy equipment in rural areas, leading to recommendations that focus on screening high-risk infants or relying only on visual assessments.

Access to phototherapy technology also varies (36). High-income settings widely use high-intensity LED phototherapy, and some have implemented home-phototherapy programs. In resource-limited settings, care may still rely on conventional fluorescent-lamp units, which provide lower irradiance and limited monitoring capacity. In these settings, exchange transfusion is often constrained by limited equipment, workforce shortages, and blood-supply constraints, leading to practical indications that differ from those in high-income countries (37).

### 4.2 Sociocultural perceptions and family role expectations

Cultural beliefs significantly influence parental understanding of hyperbilirubinemia severity, adherence to medical advice, and acceptance of community interventions (38). In regions such as India, Southeast Asia, and parts of Africa, traditional views—such as the belief that sun exposure benefits jaundiced infants or that yellow skin signifies health—are deeply ingrained, often leading to delayed medical intervention. In contrast, countries like Japan and Germany exhibit a healthcare provider-led approach, where parents are more reliant on professional guidance, resulting in higher screening compliance.

Approaches to breastfeeding policy and clinical management also vary. Some countries (e.g., France and Italy) have historically underemphasized breast milk jaundice and, in some cases, have misconstrued it as an indication for weaning. However, recent guidance from the AAP, China's 2025 guideline, and the United Kingdom's NICE emphasizes continuation of breastfeeding with appropriate monitoring. Family caregiving roles also vary. Countries such as New Zealand and Canada integrate family involvement into hyperbilirubinemia management by providing home-monitoring tools and remote guidance. By contrast, in more clinician-centered settings (e.g., China and Japan), families rely largely on clinician directives, and home phototherapy or hyperbilirubinemia monitoring devices are not yet widely implemented.

### 4.3 Technological accessibility and equipment iteration

National technological capacity substantially influences the choice of screening devices and treatment modalities, as well as the complexity and degree of personalization of guideline recommendations. In high-income settings such as the United States, the United Kingdom, and Japan, TcB devices are commonly used for initial screening. By contrast, WHO guidance and some national guidelines (e.g., India) rely primarily on TSB measurement, largely because of the high procurement and maintenance costs of noninvasive devices. In China, TcB devices are widely deployed in tertiary hospitals; however, TSB remains the primary approach in county-level and lower-tier facilities.

Regarding risk assessment tools, the Bhutani nomogram is widely utilized in countries like the United States and Canada. However, its applicability in East and Southeast Asia remains debated because of differences in population ethnicity and gestational-age distribution. China's 2025 guideline has developed locally derived dynamic curves based on national datasets. Looking ahead, some settings are exploring artificial intelligence (AI)–based risk-prediction models, which may transform risk assessment (39–41).

The integration of telemedicine also varies across settings. China's 2025 guideline proposes using mobile applications and remote platforms to support post-discharge follow-up. Japan and New Zealand have initiated pilot programs for home-based bilirubin monitoring and remote phototherapy oversight, suggesting that technological innovations may gradually narrow urban–rural gaps. By contrast, many settings in Africa and South/Central Asia currently lack the infrastructure required to implement such digital interventions at scale.

### 4.4 Drivers of guideline evolution and impact on clinical outcomes

Guideline-development methodology—ranging from expert consensus to **systematic evidence-based** frameworks—shapes both the scientific robustness of recommendations and the factors that precipitate revisions. In high-income settings, updates are frequently triggered by emerging evidence on bilirubin neurotoxicity, recalibration of treatment thresholds, advances in bilirubin measurement and phototherapy, and evolving care-delivery patterns (e.g., earlier postnatal discharge). Notably, the 2022 AAP revision explicitly acknowledges that a major challenge was redefining phototherapy and exchange transfusion thresholds, underscoring evidence-driven reassessment of intervention cutoffs (4). In the United Kingdom, NICE “exceptional surveillance” determined that CG98 requires updating, with a specific focus on TSB thresholds for initiating phototherapy and exchange transfusion—reflecting both new evidence and the need to maintain clinically safe decision thresholds at the health-system level (5). In middle-income settings (e.g., China), revision models often combine evidence synthesis with large-scale national datasets and operational considerations to support implementation across multi-tiered systems. Notably, in several African countries, the absence of unified national guidelines has been reported; hospitals may adopt external CPGs (e.g., AAP or NICE) inconsistently, potentially amplifying inter-facility variability and limiting the generalizability of single-country comparisons. By contrast, WHO-oriented guidance and some national guidelines in resource-limited settings prioritize feasibility, scalability, and minimum effective intervention packages to maximize coverage.

Whether guideline revisions translate into improved outcomes is context-dependent, and direct causal attribution remains challenging because revisions often coincide with broader system reforms and variable implementation fidelity. Nonetheless, multiple high-quality studies report outcome signals temporally associated with guideline-aligned strategies—particularly universal predischarge bilirubin screening coupled with risk-based follow-up. A large health-system study reported that universal bilirubin screening was associated with a lower incidence of severe hyperbilirubinemia, although phototherapy utilization increased (42). Population-level trend analyses further suggest that universal predischarge screening has reduced the incidence of extreme hyperbilirubinemia and the risk of kernicterus over time (43). Complementing these observational data, a randomized trial demonstrated that predischarge TcB screening reduced severe hyperbilirubinemia (risk ratio, 0.27) and jaundice-related readmissions. A subsequent systematic review and meta-analysis synthesized randomized and nonrandomized evidence evaluating universal TcB/TSB screening at discharge across multiple outcomes, including severe hyperbilirubinemia and bilirubin-induced neurologic dysfunction (44). Collectively, these findings suggest that improvements are most consistently observed when guideline updates are coupled with implementation infrastructure—screening coverage, timely access to effective phototherapy, structured post-discharge follow-up, and caregiver education—rather than textual revision alone.

## 5 The 2025 China guideline in global context

The release of the **Guidelines on the Clinical Management of Neonatal Hyperbilirubinemia (2025)** by the Chinese Medical Association represents a pivotal advance in China's approach to neonatal hyperbilirubinemia management (7). This guideline marks a shift toward a more standardized, structured, and evidence-based framework for clinical practice. Building on international evidence-based models, the guideline integrates China's multi-tiered health system and population characteristics to propose strategies across multiple domains, including screening protocols, treatment standards, high-risk population management, family-centered interventions, and digital health tools. These adaptations demonstrate strong applicability and implementation potential within China's national context.

### 5.1 Screening strategy: dual pathways of universal and enhanced high-risk screening

The guideline clearly states that all newborns should undergo bilirubin testing within 12–24 h after birth and recommends that at least two assessments be completed before discharge or within 72 h. This strategy, similar to the universal screening models endorsed by the AAP (United States) and NICE (United Kingdom), reflects a trend toward comprehensive universal screening supplemented by enhanced surveillance for high-risk infants. Given China's vast geography and pronounced urban–rural disparities, the guideline emphasizes tiered deployment of screening tools across levels of care—for example, encouraging broad TcB use in urban hospitals while allowing primary facilities to use TSB testing complemented by visual assessment for initial screening. This capability-oriented approach enhances the guideline's practicality within China and provides a transferable structural template for other settings with heterogeneous resources.

### 5.2 Treatment standards: multidimensional stratification and localized nomograms

The guideline applies a three-dimensional stratification by gestational age, postnatal age (in hours), and risk factors to guide initiation of phototherapy and exchange transfusion, and provides detailed threshold tables. For instance, preterm infants with gestational age <35 weeks have lower treatment thresholds than term infants, with earlier monitoring and intervention recommended. This logic is consistent with using the Bhutani nomogram as recommended in the AAP guideline and the hour- and risk-zone grouping in NICE. However, a notable addition in the Chinese guideline is the development of indigenous “dynamic bilirubin curves” based on domestic data, thus avoiding the adaptation issues of directly applying the Bhutani nomogram to Chinese populations. Additionally, the guideline recommends high-intensity LED phototherapy, encourages local governments to support equipment procurement, and stipulates that the need for exchange transfusion should be evaluated within 12 h of an inadequate phototherapy response, thereby reducing treatment delays—an approach that may be particularly valuable in low-resource settings.

### 5.3 High-risk population management: systematic identification and intervention pathways

The guideline elevates G6PD deficiency management to a national recommendation, emphasizing routine G6PD screening in high-prevalence regions (such as South and Southwest China) and avoidance of known hemolysis-inducing drugs. The guideline also includes dedicated sections on Rh or ABO hemolysis, infections (e.g., sepsis), and metabolic disorders. This approach goes beyond the brief mention of high-risk infants in many other national guidelines and provides more explicit pathways than WHO guidance—particularly by integrating local epidemiology with public health system responses—highlighting the potential of a guideline–screening–policy triad.

### 5.4 Management and technological integration in the home setting

To address early discharge and variable primary-care follow-up capacity, the guideline proposes a strategy of post-discharge evaluation within 48–72 h, promotion of remote bilirubin monitoring, and standardized family education. Specifically, it encourages recording bilirubin data via mobile apps or WeChat mini-programs; recommends that tertiary hospitals provide wearable TcB devices or home-phototherapy guidance for discharged infants; and includes illustrated training to help families identify warning signs such as lethargy, poor feeding, or high-pitched crying. This system is among the most advanced globally, with few countries (notably New Zealand and some regions of the United Kingdom) having achieved such deep integration of digital follow-up tools with guideline text.

China's 2025 neonatal hyperbilirubinemia management guideline, rooted in international evidence-based principles and enriched by local practice, resource adaptation, and technological innovation, offers a high-standard yet scalable model with strong primary-care orientation. Its detailed recommendations on high-risk management, remote intervention, and family support not only strengthen China's systemic response but also provide actionable lessons for global efforts to harmonize neonatal hyperbilirubinemia management.

## 6 Challenges and opportunities for global standardization of neonatal hyperbilirubinemia management

The standardization of neonatal hyperbilirubinemia management is shifting from a consensus largely derived from high-income nations to a globally integrated framework. While core principles of screening, treatment, and follow-up are converging internationally, persistent barriers in real-world settings continue to impede the uniform implementation of guidelines (45, 46). From a global health governance perspective, advancing standardization requires not only technical guideline alignment but also coordinated systems, technology transfer, cultural adaptation, and strengthened health-education capacity.

### 6.1 Key challenges

#### 6.1.1 Disparities in medical resource allocation

Medical resources remain unevenly distributed worldwide, particularly across LMICs such as India, Malaysia, and parts of Africa (47). Primary-care facilities often lack essential infrastructure, including TcB devices, standard phototherapy units, laboratory support, and follow-up systems. Although the WHO provides simplified operational templates, implementation is frequently hindered by local financial constraints and workforce limitations. Hidden costs related to equipment maintenance, replacement consumables, and health-worker training are often overlooked, leaving standardized procedures at the policy level without effective execution.

### 6.1.2 Limitations of the Bhutani nomogram

The Bhutani nomogram, widely used for risk assessment, is largely derived from U.S. cohorts and may not accurately capture bilirubin trajectories in East Asian, South Asian, or African populations. Countries like China and Japan have developed localized hour-specific bilirubin nomograms based on their own population data (48). However, many countries have yet to establish locally validated standard curves (49, 50). A globally applicable, multiethnic Bhutani nomogram remains unavailable, underscoring the need for large-scale, multi-center validation across diverse ethnic groups.

#### 6.1.3 Implementation gaps in LMICs

Despite the scientific rigor of existing guidelines, implementation in many settings depends on governmental support, insurance reimbursement mechanisms, and health-system performance incentives. In LMICs, substantial gaps persist between guideline availability and real-world implementation. Many primary-care clinicians lack systematic training and, amid heavy workloads, may have limited capacity to adhere to standardized procedures. Components such as post-discharge follow-up and family education often lack enabling mechanisms, leading to breakdowns in standardized care pathways at the last mile (51).

### 6.1.4 Cultural misconceptions and traditional practices

In some settings—particularly parts of South Asia and Africa—parents may perceive jaundice as normal or even healthy, reducing engagement in early detection (52). Traditional practices, such as sun exposure or herbal remedies, remain prevalent in some communities and can diminish acceptance of evidence-based care. Although parental education is emphasized in most guidelines, the absence of visual aids, translations into local languages, and involvement of community health workers reduces its effectiveness.

### 6.2 Strategic opportunities

#### 6.2.1 Establishing a global framework

The WHO has the political and technical capacity to promote foundational guidance globally, particularly in LMICs. Developing a Minimum Standards Framework for Neonatal Hyperbilirubinemia Management could provide a unified baseline. Collaboration with organizations such as the AAP, NICE, and the Chinese Pediatric Society could facilitate a dual-level guideline structure that combines global applicability with regional adaptation. Encouraging countries to incorporate screening, treatment, and follow-up into basic public health service packages may strengthen policy uptake and financial support.

### 6.2.2 Deploying low-cost technologies

Innovations such as affordable LED phototherapy devices, solar-powered phototherapy cribs, and portable TcB instruments are increasingly available (41, 47, 53, 54). International collaborations can reduce procurement costs, enable regional bulk purchasing, and help LMICs expand access to essential devices. China's experience suggests that provincial funding, tiered incentives, and remote maintenance mechanisms can enhance equipment availability and maintenance capacity in primary-care settings.

#### 6.2.3 Integrating digital health tools

Remote bilirubin monitoring, mobile app–based follow-up platforms, and AI-enabled early-screening models may improve efficiency across technologically disparate settings (41, 54, 55). Pilot programs in settings such as China, Australia, and New Zealand offer scalable, lower-cost models for remote management in resource-limited areas. The WHO could lead development of a Digital Health Toolkit for Jaundice, promoting national adoption and platform sharing.

#### 6.2.4 Regional collaboration mechanisms

Regions such as Asia, the Middle East, and Africa could establish multi-tiered mechanisms that combine regional consensus with national implementation. Platforms like the China-ASEAN (Association of Southeast Asian Nations) Jaundice Management Collaboration Network and the WHO African Neonatal Health Alliance can integrate guideline development, training dissemination, and data feedback. By leveraging the experience of early-adopter settings, LMICs may develop locally tailored standards aligned with global guidance (34, 56–58).

Advancing global standardization of neonatal hyperbilirubinemia management extends beyond technical guideline harmonization; it is a strategic objective for coordinating global health systems. In the context of resource disparities, cultural diversity, and institutional differences, a comprehensive approach encompassing technology simplification, policy coordination, digital support, and international collaboration is essential to achieve consensus standards with local adaptation.

## 7 Strategic recommendations for harmonizing neonatal hyperbilirubinemia management globally

### 7.1 Establishing a global governance and support framework

Global standardization of neonatal hyperbilirubinemia management requires moving beyond harmonizing guideline text to establishing a comprehensive governance framework centered on equitable access to screening, standardized treatment, family engagement, and sustained follow-up.

#### 7.1.1 Adopting a dual-structured guideline model

At present, international guidance reflects the coexistence of high-standard and simplified, pragmatic approaches (59). The WHO should lead efforts to integrate established content from the AAP, NICE, and the Chinese Pediatric Society into a global neonatal hyperbilirubinemia recommendation system with a universal core and a regionally adaptive layer. The universal core should include basic screening principles, prioritization of phototherapy, and minimum safety criteria for exchange transfusion applicable across settings. The adaptive layer would encourage countries or regions to develop dynamic curves, follow-up mechanisms, and family education models tailored to local resources and population characteristics. This model can maximize implementation flexibility and cultural acceptability while safeguarding core safety standards.

#### 7.1.2 Enhancing global access to technology

WHO and the United Nations Children's Fund (UNICEF) should leverage multilateral procurement platforms to coordinate device dissemination and maintenance mechanisms in LMICs. The rollout of low-cost, modular TcB meters and phototherapy devices should be tailored to primary care. International bulk procurement and national co-financing could lower acquisition barriers. Belt and Road newborn-health cooperation mechanisms could support technology transfer, training, and remote maintenance, facilitating dissemination of validated low-cost solutions from China, ASEAN, and other settings (47).

#### 7.1.3 Localizing data and risk assessment

The racial and ethnic limitations of risk-assessment tools such as the Bhutani nomogram remain a key technical challenge for global standardization (49, 60). Countries should be encouraged to establish locally derived, hour-specific bilirubin curves. Support should be provided to develop multiethnic risk-prediction models incorporating postnatal age (in hours), genetic background, and feeding patterns. In parallel, a global jaundice data-sharing platform could facilitate transparent research collaboration, model sharing, and retrospective validation—thereby improving the generalizability and accuracy of these tools.

#### 7.1.4 Building family-centered, community-based follow-up

The family is a critical setting for hyperbilirubinemia management; guidelines should explicitly address family education, remote management, and community follow-up mechanisms (61). All countries should incorporate a 48–72-hour post-discharge re-evaluation mechanism in their guidelines, linked to coordinated obstetric–pediatric care. Countries could also draw on experience from China and New Zealand by developing family bilirubin record cards, mini-programs, and teleconsultation platforms. In LMICs, strengthening community health-worker networks could enable jaundice recognition, follow-up registration, and education—helping to address workforce gaps.

#### 7.1.5 Integrating metrics into national health performance

A key step in moving from guideline recommendations to policy realization is inclusion in national health targets and fiscal performance metrics. Countries should include indicators such as screening coverage, timely phototherapy, and follow-up completion in core health-ministry performance frameworks. Financial incentives and data feedback can support continuous quality improvement in hospitals and primary-care units. The WHO and the World Bank could also recognize and incentivize high-performing countries through global maternal and child health performance funds.

### 7.2 Strengthening localization and community-based implementation

Beyond policy harmonization, specific research and technological gaps must be addressed to operationalize these guidelines.

#### 7.2.1 Recalibrating diagnostic tools

Given the variable performance of TcB devices across skin tones, future studies should develop and validate skin-pigmentation correction algorithms. This is critical to reducing measurement bias in African and South Asian populations.

#### 7.2.2 AI-driven risk stratification

To address the limitations of the United States-centric Bhutani nomogram, international collaborations could leverage federated learning to develop dynamic, multivariable risk-prediction models applicable across diverse genetic backgrounds.

#### 7.2.3 Standardizing low-resource interventions

For off-grid regions, filtered sunlight phototherapy (FSPT) could be formalized. Clear technical specifications (e.g., ISO standards) for FSPT devices are needed to ensure safe ultraviolet filtration and prevent hyperthermia, enabling formal inclusion in guidance for resource-limited settings.

#### 7.2.4 Validating mHealth screening

To address workforce shortages in LMICs, randomized controlled trials are needed to evaluate task-shifting models in which community health workers use smartphone-based bilirubin-screening apps to triage high-risk infants.

In summary, global standardization of neonatal hyperbilirubinemia management cannot rely solely on medical consensus; it also requires coordinated political commitment, technical alignment, and societal engagement. Future efforts should emphasize consensus first, adaptive tolerance, technological empowerment, and governance collaboration—promoting integrated system building and practical implementation while respecting real-world differences. Only through such an approach can early screening, timely treatment, and effective control be achieved globally, thereby reducing the burden of kernicterus and bilirubin-associated morbidity and mortality.

## 8 Conclusion

Neonatal hyperbilirubinemia is one of the most common perinatal conditions worldwide. Although it is usually self-limiting, absent screening or delayed intervention can lead to acute bilirubin encephalopathy and, in severe cases, kernicterus—making it a major preventable cause of neurodevelopmental impairment. International guidelines have shifted from experience-based approaches to evidence-based protocols for screening, treatment, and post-discharge follow-up. This narrative review systematically compares guidance from the AAP, the United Kingdom's NICE, the WHO, and China across key domains, including universal screening coverage, diagnostic approaches, phototherapy thresholds, exchange transfusion criteria, high-risk identification, and family support. This analysis highlights a central pattern in global neonatal hyperbilirubinemia management: conceptual convergence alongside structural divergence. However, real-world barriers such as uneven resource distribution, cultural differences, poor nomogram adaptability, and insufficient institutional enforcement continue to constrain the global expansion of standardized care pathways. Future efforts must leverage WHO and regional health organizations to advance a dual-layer guideline structure, promote low-cost device dissemination, enable locally adapted AI-driven nomograms, establish community-oriented family follow-up systems, and embed jaundice management performance metrics into national policy frameworks. Standardization of neonatal hyperbilirubinemia care should not stop at harmonizing guideline content but should progress toward aligned implementation across health systems, sociocultural contexts, and resource settings. Only through such efforts can the public health goals of early screening, timely treatment, and effective control be achieved—ensuring equitable, safe, and accessible care for newborns worldwide.
